# Seawater drowning induced acute lung injury: new insights from novel mouse models and micro-CT imaging

**DOI:** 10.1515/med-2026-1510

**Published:** 2026-08-24

**Authors:** Jinxia Liu, Chunsun Li, Zhen Yang, Yuanhui Wei, Zhixin Liang, Xiuqing Ma, Shangshu Liu, Jiabo Ren, Zhenfei Mo, Yue Yin, Zirui Wang, Liangan Chen

**Affiliations:** Medical School of Chinese PLA, Beijing, P.R. China; Department of Pulmonary and Critical Care Medicine, Chinese PLA General Hospital, Beijing, P.R. China; School of Medicine, Nankai University, Tianjin, P.R. China

**Keywords:** mouse, seawater drowning induced acute lung injury (SWD-ALI), micro-computed tomography (micro-CT)

## Abstract

**Objectives:**

Seawater drowning causes severe lung and alveolar damage, easily inducing acute respiratory distress syndrome with high early mortality. Currently, standardized assessment and treatment approaches for seawater drowning-induced acute lung injury (SWD-ALI) are lacking. This study aimed to explore the lung damage mechanism of seawater drowning and provide early diagnostic and clinical therapeutic references for SWD-ALI.

**Methods:**

A novel mouse model of SWD-ALI was established for the first time via the laryngoscopic endotracheal injection (LEI) method. Multiple indicators including lung histopathology, survival rate, bronchoalveolar lavage fluid, lung coefficient and micro-CT were detected in strict accordance with the latest animal acute lung injury testing standards.

**Results:**

Obvious exudative lesions in lung tissues were detected by CT as early as 0.5 h after seawater-induced injury, enabling early identification of severe acute lung injury.

**Conclusions:**

Lung CT can realize early diagnosis of SWD-ALI. This finding is critical for guiding clinical treatment and reducing the mortality of seawater drowning patients.

## Highlights

1. Establishment of a SWD-ALI mouse model
2. The SWD-ALI mice model was prepared by LEI method
3. Micro-CT accurately evaluated the injury degree and location of SWD-ALI.

## Introduction

Drowning is defined as submergence in water and asphyxiation due to water inhalation into the lungs or pure asphyxia, and it is a major global public health and safety issue [1]. The Global Burden of Disease study identified that drowning is one of the most common causes of unintentional death, accounting for approximately 500,000 deaths worldwide each year [2]. Seawater is a hypertonic liquid containing 3.5 % sodium chloride, large amounts of calcium and magnesium salts [3]; hypertonic seawater exerts a strong damaging stimulus on the lungs and alveoli, attracting fluid from pulmonary capillaries into the alveoli to cause hemoconcentration, hypovolemia, and pulmonary edema. In addition, seawater contains abundant microorganisms such as bacteria [4], which can lead to severe secondary infections. During the progression of seawater drowning (SWD), complications such as respiratory tachycardia, hypoxemia, disseminated intravascular coagulation, and acute renal failure may occur, and acute lung injury (ALI) stands out as its most prevalent and life-threatening complication [5]. Due to the lack of effective targeted treatments, SWD-induced ALI (SWD-ALI) has an extremely high early mortality rate, making early and accurate assessment of ALI severity pivotal for guiding clinical intervention and reducing mortality [6]. Traditional methods for establishing SWD-ALI mouse models include Direct Drowning (DD), Nasal Drip (ND), and Neck incision endotracheal injections (NEI), all of which have obvious limitations: DD exhibits a very high mortality rate and substantial individual differences; ND has uncontrollable inhalation dose and poor model stability;NEI requires invasive neck surgery, causes severe systemic inflammatory response, and has a long anesthesia time [7], [8], [9], [10]. Such limitations restrict the in-depth exploration of SWD-ALI pathogenesis and the development of targeted therapeutic drugs.

Computed tomography (CT), such as micro-CT, has the advantages of high sensitivity, rapid detection, and non-invasiveness, and has been widely used in the diagnosis and evaluation of respiratory diseases [11], 12]. Mobile CT enables more convenient bedside evaluation for critically ill patients [13], [14], [15]. However, there is a lack of robust evidence for the establishment of a stable SWD-ALI model via an optimized technique, and whether early micro-CT can accurately assess SWD-ALI severity and guide clinical intervention remains unclear.

The lack of a stable, reproducible SWD-ALI mouse model and the unclear value of early micro-CT in injury assessment are the key bottlenecks restricting SWD-ALI research and clinical translation. Based on the clinical characteristics of seawater inhalation via the upper respiratory tract, we designed the Laryngoscopic Endotracheal Injection (LEI) method for SWD-ALI model establishment, which recapitulates clinical seawater inhalation and circumvents the limitations of traditional methods. We hypothesize that the LEI method can stably establish a SWD-ALI mouse model with moderate mortality and controllable injury severity, and micro-CT enables accurate early identification of the lesion location and severity in SWD-ALI, thus offering a reliable foundation for clinical guidance. The core research questions of this study are: (1) Can the LEI‑based artificial seawater injection establish a stable and reliable SWD-ALI mouse model? (2) Can micro-CT accurately evaluate the degree of lung injury in SWD-ALI mice and provide an early basis for treatment selection?

## Materials and methods

### Reagent

Artificial seawater was prepared with reference to the table of major components of seawater along the southeast coast provided by the Third Research Institute of the Bureau of Oceanography of China [16], with the exact mass to volume concentrations of major salts as follows: NaCl 35.0 g/L, MgCl_2_ 5.0 g/L, MgSO_4_ 7.0 g/L, CaCl_2_ 1.1 g/L, KCl 0.7 g/L, NaHCO_3_ 0.2 g/L, NaBr 0.1 g/L. Sodium chloride was obtained from Sinopharm Chemical Reagent Co., Ltd (Shanghai, China); magnesium chloride, magnesium sulfate, calcium chloride, potassium chloride, sodium bicarbonate and sodium bromide were all purchased from Beijing Sunshine Yingrui Biotechnology Co., Ltd (Beijing, China); phosphate buffer was supplied by Gibco (New York, NY, USA); and the BCA assay kit and Trypan blue were sourced from Thermo Fisher Scientific (Massachusetts, USA).

### Animal studies

Healthy male C57BL/6 mice (6–8 weeks old, body weight 20–25 g) were provided by the Experimental Animal Center of the Chinese PLA General Hospital. After 1 week of acclimatization under standard laboratory conditions (temperature 22–25 °C, 12 h light-dark cycle, free access to food and water), mice were randomized into experimental groups using a computer-generated random numbers (SPSS 27.0) by an independent experimenter not involved in subsequent model preparation or outcome assessment; the randomization sequence was concealed in sealed envelopes until group allocation to ensure allocation concealment. The baseline body weight and general activity status of mice in each group were assessed and compared prior to modeling, with no significant intergroup differences observed (*p*>0.05).

The sample size for experiments was pre-determined via power analysis using G*Power 3.1 software, confirming that 5–7 mice per group were sufficient to detect significant intergroup differences for primary outcomes including survival rate, lung coefficient, and percentage of injured lung area on micro‑CT – a sample size consistent with our previously published SWD-ALI research with reproducible and statistically significant results [17]. Seven mice from each group were used for survival analysis, with death or 24 h survival set as the termination event, and survival monitored every 4 h over the 24‑hour observation period.

### DD: direct drowning

Mice were individually placed in container filled with artificial seawater (6 cm depth, 25 °C). Mice were rapidly retrieved after 25 s and dried with filter paper. Vital signs, physical state, and survival status were closely observed and documented.

### ND: nasal drip

The operator stabilized the mouse’s lower jaw with the left thumb and forefinger, supporting the torso with the remaining fingers and palm to maintain an upright position with head hyperextension. A pipette was used to instill 4 mL/kg artificial seawater into the nasal cavity, and the mouse was vertically rotated for 15 s following instillation to facilitate uniform pulmonary distribution of seawater. Seawater inhalation, physical state, and survival were recorded.

### NEI: neck incision endotracheal injection

Mice were fixed in a supine position on a surgical board at a 60–70° angle to the ground. The anterior cervical skin and fascia were incised, muscle tissue was separated to expose the trachea, and a 1 mL syringe was inserted into the trachea to inject 4 mL/kg artificial seawater at a uniform rate over 2 min. The mouse was rotated vertically for 15 s after injection. Tracheal fluid changes, respiration, cough reflex, cyanosis, and survival were observed and recorded.

### LEI: laryngoscopic endotracheal injection

Mice were secured in a supine position on a surgical board at a 60–70° angle to the ground. Isoflurane inhalation anesthesia was used for all invasive procedures (induction: 3–5 L/min for 1–2 min, maintenance: 1–2 L/min for ≤5 min) with strict control of anesthesia duration to avoid respiratory or cardiac dysfunction. The operator held a laryngoscope with the left hand, inserted it into the oral cavity via the right buccal commissure, and gently retracted the mouse’s tongue with the right hand. The laryngoscope was used to lift the epiglottis, revealing the inverted triangular white cartilage that opens and closes with respiration. 4 mL/kg artificial seawater was injected via a microsyringe during cartilage opening, and the mouse was vertically rotated for 15 s post-injection using the identical procedure described above. Respiration, cough reflex, cyanosis, and survival were observed and recorded.

The 2 mL/kg, 4 mL/kg, and 8 mL/kg artificial seawater doses were selected based on clinical translation and pre-experimental screening: pre-experiments confirmed that a 1 mL/kg dose induced no significant lung injury, while a 10 mL/kg dose resulted in 100 % mortality within 4 h. Thus, 2,4,8 mL/kg were set as low-medium-high doses to reflect a dose-dependent SWD-ALI response, with the 4 mL/kg dose consistent with our published SWD-ALI model for inducing stable moderate lung injury [17].

### Lung coefficient

Mice body weights (B) were recorded before sampling by an investigator blinded to group allocation, and the wet weights (W) of the lungs were measured immediately after sampling. Lung coefficient was calculated as W/B ×100 %, a validated index for assessing the severity of pulmonary edema.

### Mouse micro-CT imaging

Mice were anesthetized with isoflurane. Subsequently, the mice were positioned in the CT device and scanned using the Quantum FX microimaging system (PerkinElmer, USA). Each mouse was scanned at 70 kV, 88 μA and 36 mm field‑of‑view (FOV) for 4 min. All micro-CT image analyses were performed in a retrospective double-blinded manner by two independent radiologists with expertise in small animal imaging. Scan data were analyzed using Analyze 12.0 software with standardized horizontal settings. 3D-Slicer software was used to segment the lung and the damaged area, and Python was used to calculate the damaged area, percentage of damaged area, and average gray value. Based on the 95 % reference interval of the control group, early micro-CT diagnostic criteria for murine SWD-ALI were established accompanied by focal or diffuse alveolar exudation. Quantitative data of average gray value and damaged area ratio for all time points (0.5 h, 2 h, 3 h, 6 h, 24 h) were calculated and presented as a time-course to show the dynamic changes of lung injury.

### H&E staining of lung tissues

The lung tissues were fixed with 4 % paraformaldehyde, embedded in paraffin, and cut into 5 μm sections. Subsequently, H&E staining was performed, and photographs for damage assessment by a pathologist blinded to group allocation. Lung injury severity was assessed using a semi-quantitative pathological scoring system, which evaluated alveolar edema, hemorrhage, inflammatory cell infiltration, and alveolar architectural disruption.

### Bronchoalveolar lavage fluid (BALF) analysis

After cervical tracheostomy, tracheal intubation and fixation were performed in mice. Bronchoalveolar lavage was conducted three times using 0.9 mL ice-cold 0.9 % PBS each time, with two repeated aspirations for fluid collection. The total lavage volume was 2.7 mL, and the recovery rate exceeded 80 %. The centrifugal pellet was resuspended in PBS. Then, 10 μL of the cell suspension was mixed with 10 μL AOPI, loaded into a counting chamber, and the total cell number was determined with a cell counter. A 20 μL aliquot of each sample was used for H&E staining, and the percentage of neutrophils was calculated by an analyst blinded to group allocation.

### Detection of protein concentration in BALF

The protein concentration in BALF supernatant was quantified using the Bicinchoninic Acid (BCA) assay, and the absorbance was measured at 562 nm with a microplate reader. The protein concentration of the sample was then calculated against the standard curve.

### Statistical methods

SPSS 27.0 software was used for statistical analysis. A Blinding design was implemented for all experimental procedures: analysts of micro-CT images, pathological sections, and BALF cell counting were blinded to group allocation, with all samples coded with random numbers (decoded only after all data collection). Data were expressed as the mean ± standard error of the mean (SEM), and normality was assessed accordingly. One-way ANOVA was applied for comparisons among multiple groups with normal distribution and homogeneous variance, while the Kruskal-Wallis H test was used for non-normally distributed data. Survival curves were constructed by the Kaplan-Meier method and compared via the Log-rank test. Pearson correlation analysis was used to evaluate the correlation between micro-CT damaged area percentage and pathological total injury score. *p*<0.05 was considered statistically significant.

### Ethical approval

All experimental protocols were approved by the Experimental Animal Welfare Ethics Committee of Chinese PLA General Hospital (Approval No. 2024-X20-69). All animal experiments were performed in strict accordance with the *Guide for the Care and Use of Laboratory Animals* (8th Edition, NIH) to minimize animal suffering and ensure humane animal care.

## Results

### LEI method can stably establish a mouse SWD-ALI model

**Figure 1:**
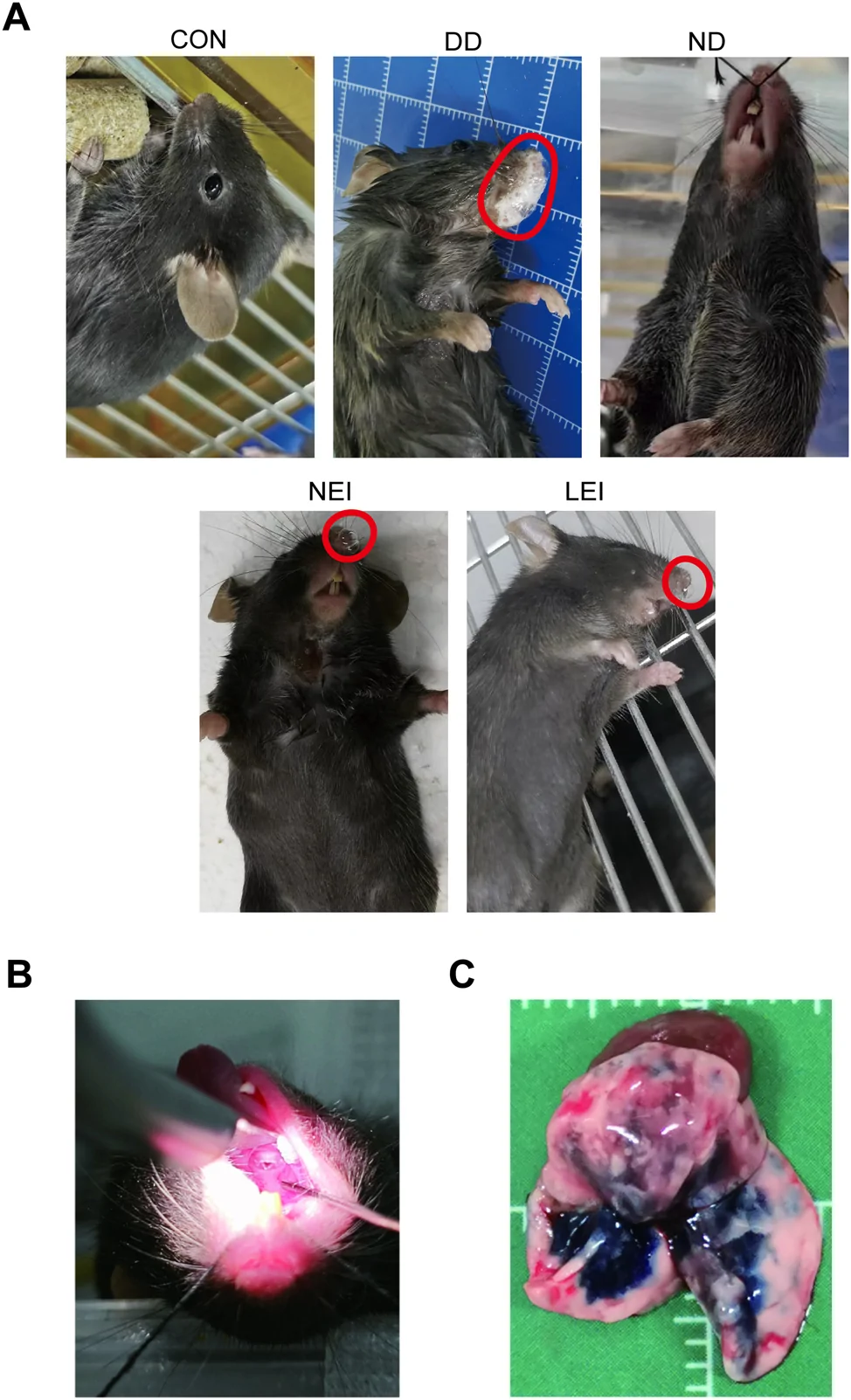
Different methods of preparation of SWD-ALI. Notes: A state of SWD-ALI mice prepared by different methods (the red line area is secretions); B laryngoscopic picture of the vocal cords in mice; C localization of trypan blue by LEI method. n=7 per group.

Four methods were used to establish the C57BL/6 mouse SWD-ALI model, with the post-modeling status of mice shown in Figure 1A (n=7 per group). Mice in the control group (CON) were more active, with without dyspnea, choking and oronasal secretion overflow from the mouth and nose. The mice in the DD group showed severe dyspnea, and there were a lot of white foam discharge overflow, accompanied by obvious hypothermia and cyanosis. Mice in the ND group showed partial dyspnea, with no secretion overflow from the mouth and nose. Mice in the LEI group and the NEI group had obvious choking and dyspnea, and there were clear white foamy secretion overflowing from the mouth and nose, and mild to moderate cyanosis. In the transoral laryngoscopy, the vocal folds of the mice were seen to be white and inverted triangular (Figure 1B), opening and closing synchronously with respiration. Trypan‑blue staining in the LEI group precisely identified the injection site (Figure 1C). CON mice underwent the same isoflurane anesthesia procedure (without seawater injection) for micro-CT and lung coefficient detection, confirming that isoflurane anesthesia had no adverse effects on murine lungs and excluding anesthesia as a confounding factor.

**Figure 2:**
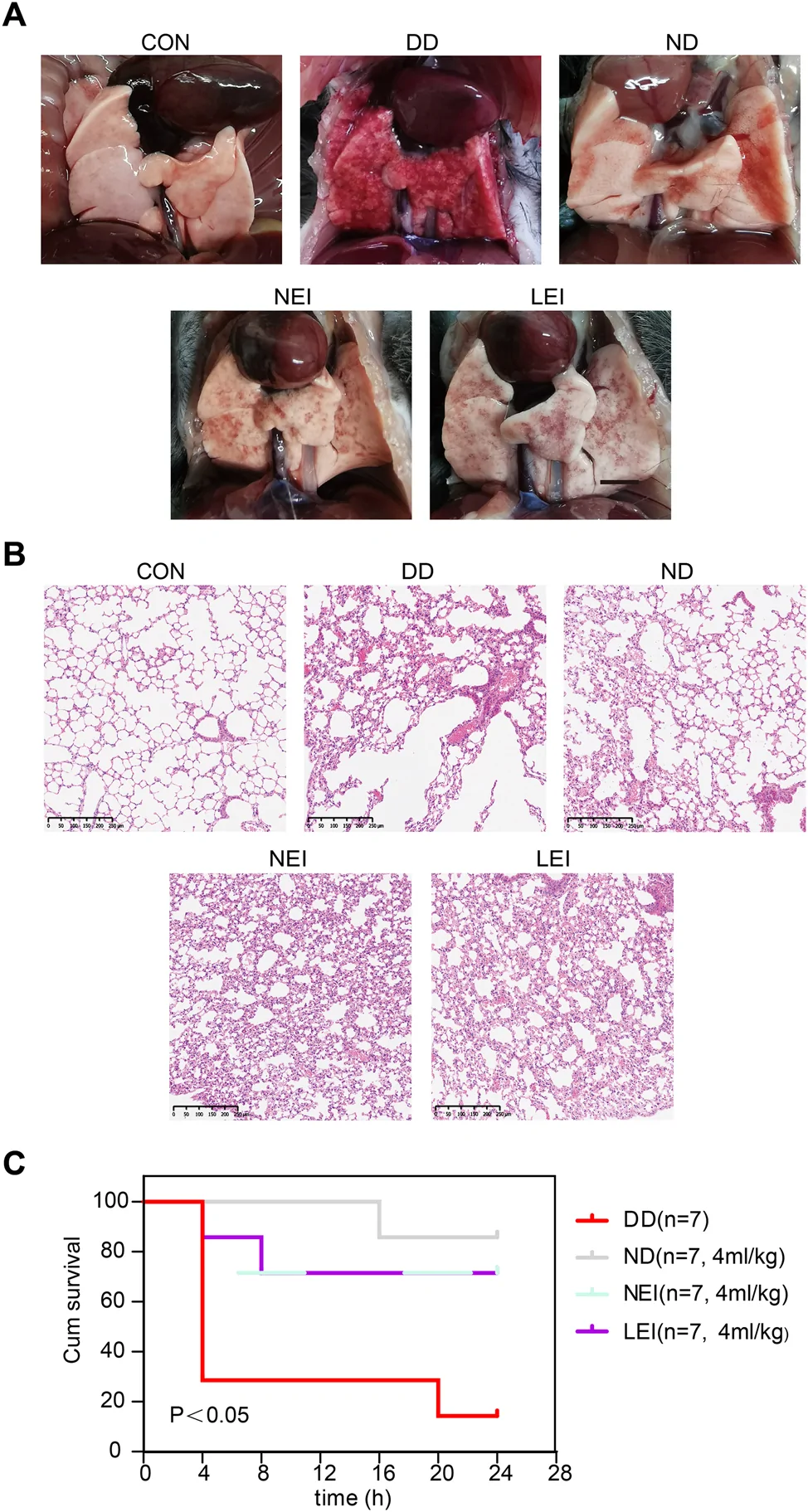
Stabilization of modeling injury in a SWD-ALI mice model prepared by LEI. Notes: A representative lung tissues sections from each group of mice; B representative H&E-stained images from each group of mice; C 24 h survival analysis modeled by different methods. n=7 per group, *p<0.05 (log-rank test).

Model injury was verified through pathological examination and survival analysis (Figure 2A and B, n=7 per group). Lung tissues of CON group mice were pale pink, and there was no hemorrhage or edema in H&E staining. The lung tissues of DD group showed severe swelling and congestion changes, and H&E staining showed localized lung atelectasis, hemorrhage, edema, inflammatory cell exudation, with obvious alveolar rupture in some areas. The lung tissues of ND group showed local redness and swelling, and the pathological manifestations were focal relatively mild edema with less hemorrhage. NEI and LEI group lung tissues exhibited varying degrees of swelling and hemorrhage, H&E staining showed inflammatory cell exudation and pulmonary hyaline membrane formation – ”key pathological features of clinical SWD-ALI” [7]. Survival analysis of different modeling methods (Figure 2C, n=7 per group) suggested that five mice died in the DD group at 4 h, and one died at 20 h. There were no deaths in the ND group within 24 h. Two mice died in the NEI group at 4 h, one died in the LEI group at 4 h and one at 8 h. Differences in survival rates among the four groups were statistically significant (p<0.05), with the LEI group exhibiting a moderate mortality rate that avoided the excessively high mortality of the DD group and mild injury of the ND group, confirming model stability.

### LEI of 4 mL/kg artificial seawater induced stable moderate ALI in mice

Based on the above results, the LEI method was selected for further exploration of different artificial seawater doses, with mice in different dose groups showing distinct dose-dependent pathological and clinical states (n=7 per group). Mice in the 2 mL/kg group were the most active, with the mildest respiratory distress and no obvious secretion from the mouth and nose. Mice in 8 mL/kg group had obvious choking and respiratory distress, with more white foamy secretion overflowing from the mouths and noses. The cyanosis was visible, and some of them showed hypothermia and even died. The performance of 4 mL/kg mice was between two groups, with moderate respiratory distress and foamy secretions, consistent with stable moderate ALI.

**Figure 3:**
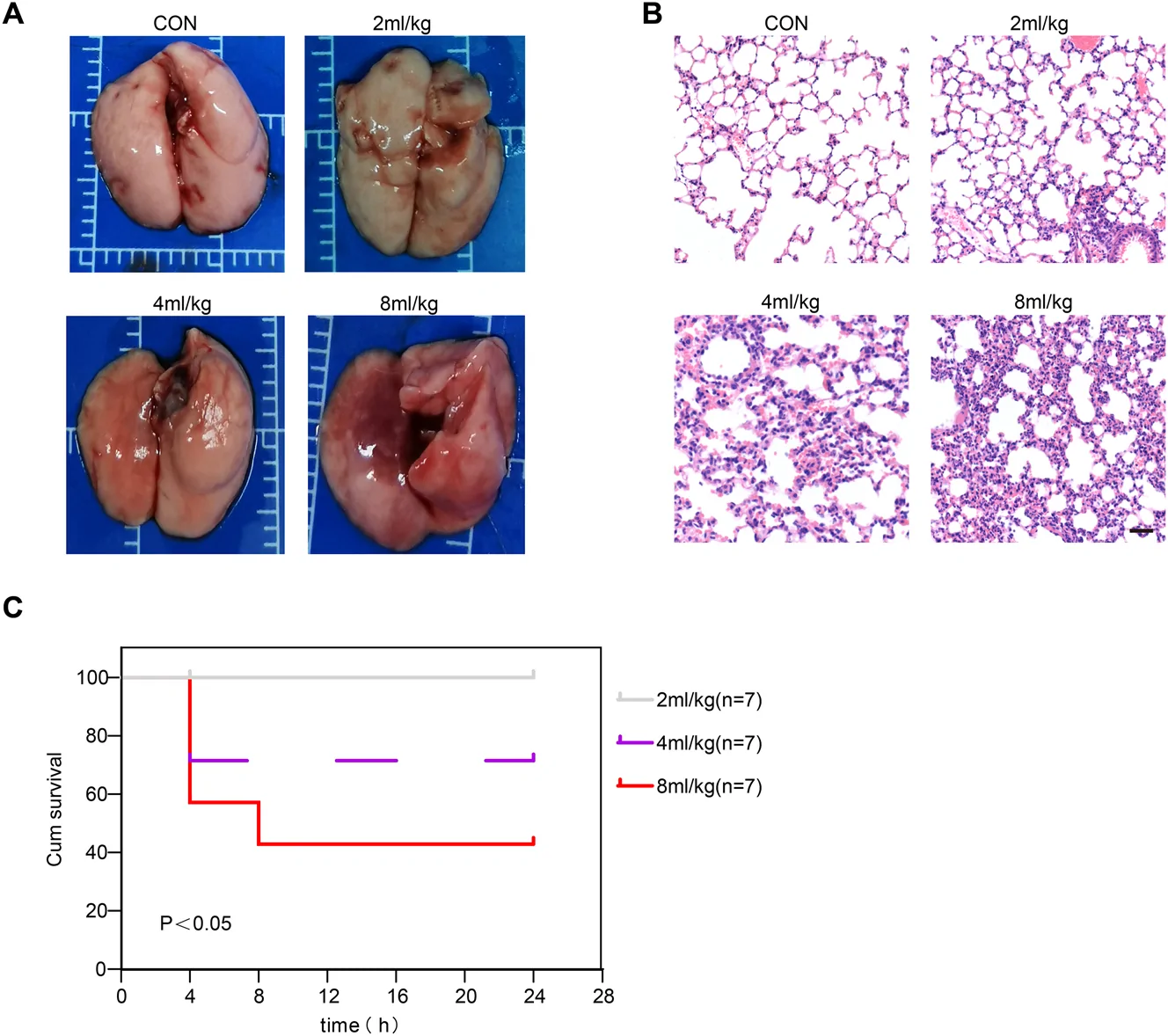
The LEI method of artificial seawater 4 mL/kg injury was relatively stable. Notes: A representative lung tissues images from each group of mice; B representative H&E-stained images from each group of mice; C 24 h survival analysis modeled by different doses. n=7 per group, *p<0.05 (log-rank test).

Lung tissues (Figure 3A and B, n=7 per group) of 2 mL/kg group mice showed local scattered redness and swelling, and the pathological manifestation showed focal and relatively mild edema with less hemorrhage. Lung tissues of 4 mL/kg group mice exhibited obvious alveolar and interstitial swelling, hemorrhage, and inflammatory cell infiltration – consistent with moderate ALI and stable injury characteristics suitable for subsequent experimental analysis. In contrast, 8 mL/kg group mice had swollen and congested lungs, H&E staining showed obvious atelectasis, pulmonary hyaline membrane formation, alveolar and interstitial inflammation and bleeding, and severe pulmonary edema. Survival curves of mice exposed to different doses of seawater inhalation were analyzed (Figure 3C, n=7 per group). No mortality was observed in the 2 mL/kg group during the observation period. In the 4 mL/kg group, 2 mice died within 4 h. Three mice in 8 mL/kg group died within 4 h period, one mouse died within 8 h, and the survival rate in the 24 h period was lower than 50 %. Differences in survival rate among the three groups were statistically significant (p<0.05), confirming the dose-dependent effect of artificial seawater on SWD-ALI induction via LEI.

### Early lung imaging damage can be easily detected in SWD-ALI mice after LEI artificial seawater

In order to observe the changes of lung injury at different times after seawater inhalation, the clinical manifestations, imaging, pathology, and BALF changes of mice after injury were compared (micro-CT: n=5 per time point; pathology/BALF: n=6 per time point). Firstly, the clinical manifestations of the mice in the 0.5 h and 2 h groups showed white or transparent secretions from the mouth and nose, accompanied by different degrees of choking and coughing, dyspnea, wet crackles, cyanosis, obvious reduction of activity, unkempt hair and lack of luster. Compared with two groups above, the mice in the 6 h group showed obvious reduction of secretions from the mouth and nose, but the dyspnea was more obvious, accompanied by varying degrees of cyanosis, and there was an obvious reduction of activity, reduced feeding, more unkempt hair and lack of luster. Mice in 1d group had no obvious oral and nasal secretions, still had dyspnea, less activity and feeding, unkempt fur and dull luster. Mice in 3d, 7d and 28d groups showed gradual clinical improvement but still had activity-induced dyspnea and poor motor flexibility – a recovery course consistent with clinical SWD-ALI patients [5].

**Figure 4:**
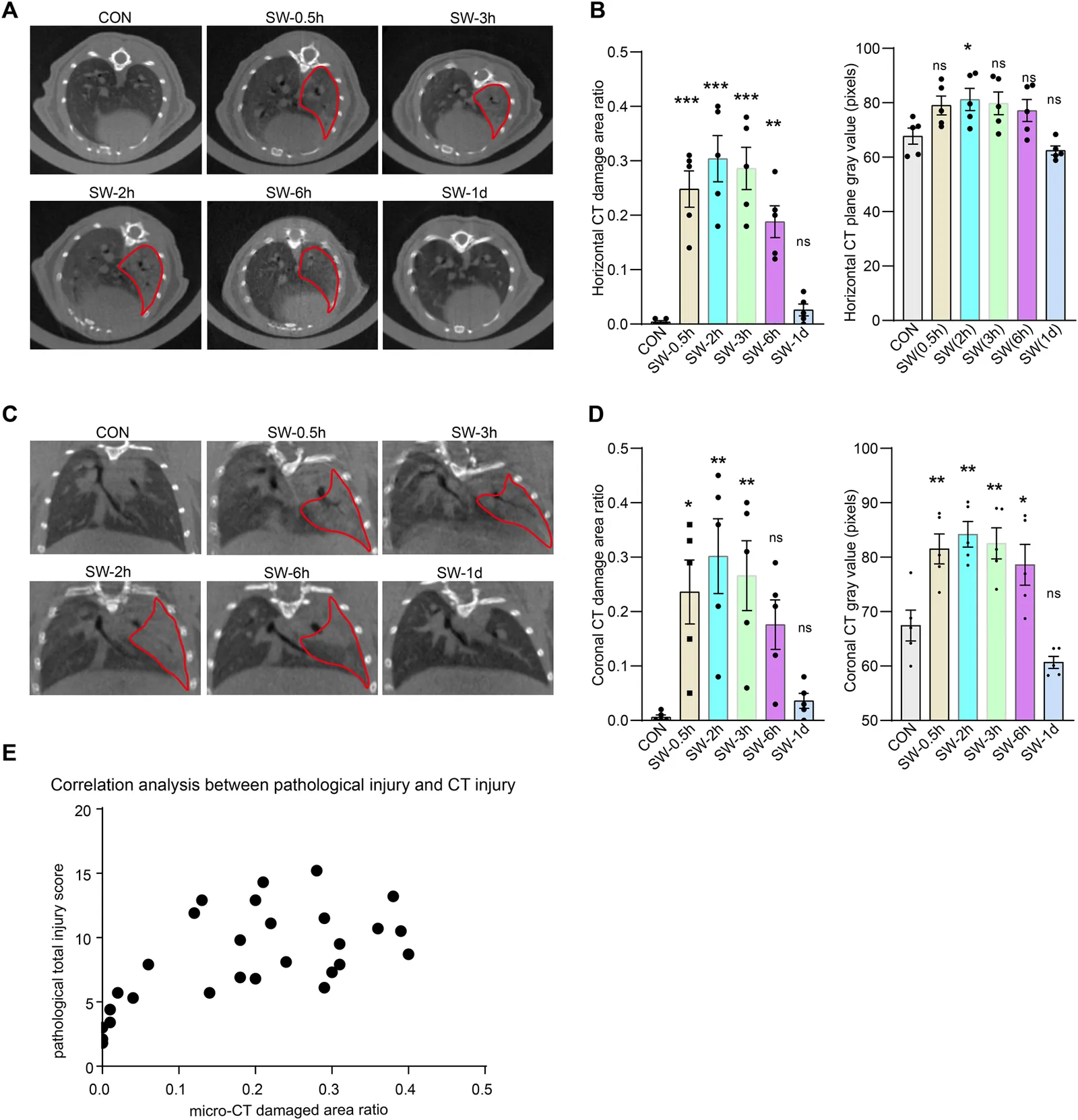
Lung CT enables rapid assessment of SWD-ALI damage at an early stage. Notes: A representative micro-CT horizontal images of different times after modeling in mice (the red line area is the damage area); B damage area ratio and average gray value of horizontal lung CT, n=5, *p<0.05, **p<0.01, ***p<0.001; C representative Micro-CT coronal images of different times after modeling in mice (the red line area is the damage area); D damage area ratio and average gray value of coronal lung CT, n=5, *p<0.05, **p<0.01; E correlation scatter plot of micro-CT damaged area ratio and pathological total injury score (Pearson r=0.64, p<0.001).

Secondly, Micro-CT imaging was performed to assess lung injury, with similar expiratory phases selected for all mouse lung scans to ensure consistency (Figure 4A, 4C). CON group horizontal CT images showed uniform low-density shadows (gas-filled alveoli) with no exudation. In 0.5 h group had extensive exudative changes in the left lung with reduced gas content and mild exudation in the right lung. The 2 h group exhibited the “most severe exudative changes (imaging peak)”, with significantly aggravated left lung exudation and new small amounts of exudation in the right lung, consistent with early “white lung” changes in clinical ALI/ARDS [18]. The 3 h and 6 h groups showed gradual improvement in lung imaging with progressive absorption of exudate, and the 24 h group had near-normal lung imaging with minimal residual exudation. Damage area ratio and average gray value in horizontal (Figure 4B) and coronal (Figure 4D) CT images for all time points (0.5 h, 2 h, 3 h, 6 h, 24 h) were quantified; both indices were significantly increased in the 2 h group (p<0.05), and gradually decreased at 3 h and 6 h, returning to near normal at 24 h. A significant positive correlation was found between micro-CT damaged area ratio and pathological total injury score (Pearson r=0.64, p<0.001) (Figure 4E), validating the accuracy of micro-CT in quantifying SWD-ALI severity.

**Figure 5:**
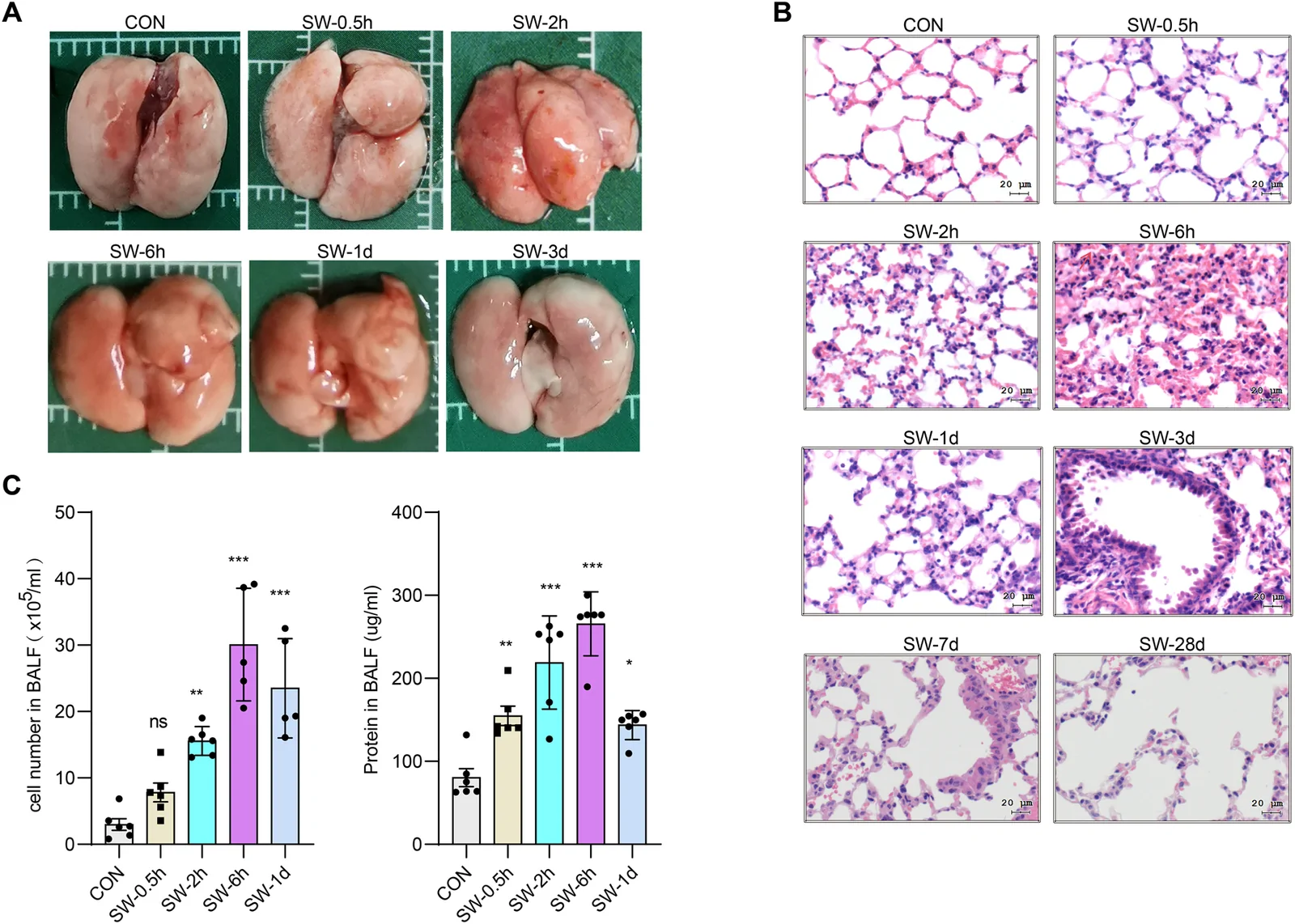
The SWD-ALI mouse model was prepared by LEI and the damage was obvious at 6 h. Notes: A The representative lung tissue images at different time points after modeling; B representative H&E staining images at different time points after modeling; C compared with the CON group, the total cell counts and total protein concentration in BALF were determined at different time points after modeling, n=6, ^ns^P>0.05, *p<0.05, **p<0.01, ***p<0.001.

Thirdly, the lung tissues (Figure 5A, n=6 per time point) of mice in the 0.5 h group showed obvious edema in the lungs, with relatively little hemorrhage. However, the central part of the lung was bleeding (bright red), and there was still significant edema in the 2 h group. The edema in the lung tissues of mice in the 6 h group was slightly improved, but obvious hemorrhagic manifestations were seen, which were mainly centrally distributed (“pathological peak”). The hemorrhage and edema were partially absorbed in the lung tissues of mice in 1d group. Mice lung tissues of 3d group showed edema was absorbed, but stale hemorrhage was visible, and the damage site was dark red, mainly centrally distributed.

Then, the mice inhaled seawater for different periods of time showed different lung pathological changes (Figure 5B, n=6 per time point). The lungs of mice in the 0.5 h group showed local edema, increased inflammatory cells, local alveolar structure disappeared. The lungs of mice in the 2 h group showed part of the interstitial edema, hemorrhage, alveolar collapse, inflammatory cells and erythrocytes had aggregation. The lungs of mice in the 6 h group showed obvious alveolar collapse, disappearance of local alveolar structures, alveolar and interstitial edema, hemorrhage, and severe inflammatory cell exudation. In the 1d group, alveolar and interstitial hemorrhage and edema were slightly improved, the alveoli were partially expanded, and different degrees of inflammatory cells were exudated. In the lungs of mice in the 3d group, some epithelial cells in the small bronchus were detached and a few macrophages were observed. The lung injury was recovered in the 7d group, but the small bronchial blunt rupture was obviously dispersed, and the lumen contents were significantly reduced compared with those in the 3d group. Some of the subpleural lung tissues in the 28d group showed alveolar rupture and hyperventilation. Finally, total protein concentration and total cell number (Figure 5C, n=6 per time point) in BALF were raised post-inhalation seawater, and those in the 6 h group were the highest among the time points measured, consistent with the pathological peak of lung injury.

### The SWD-ALI model in mice conformed the criteria for animal ALI

**Figure 6:**
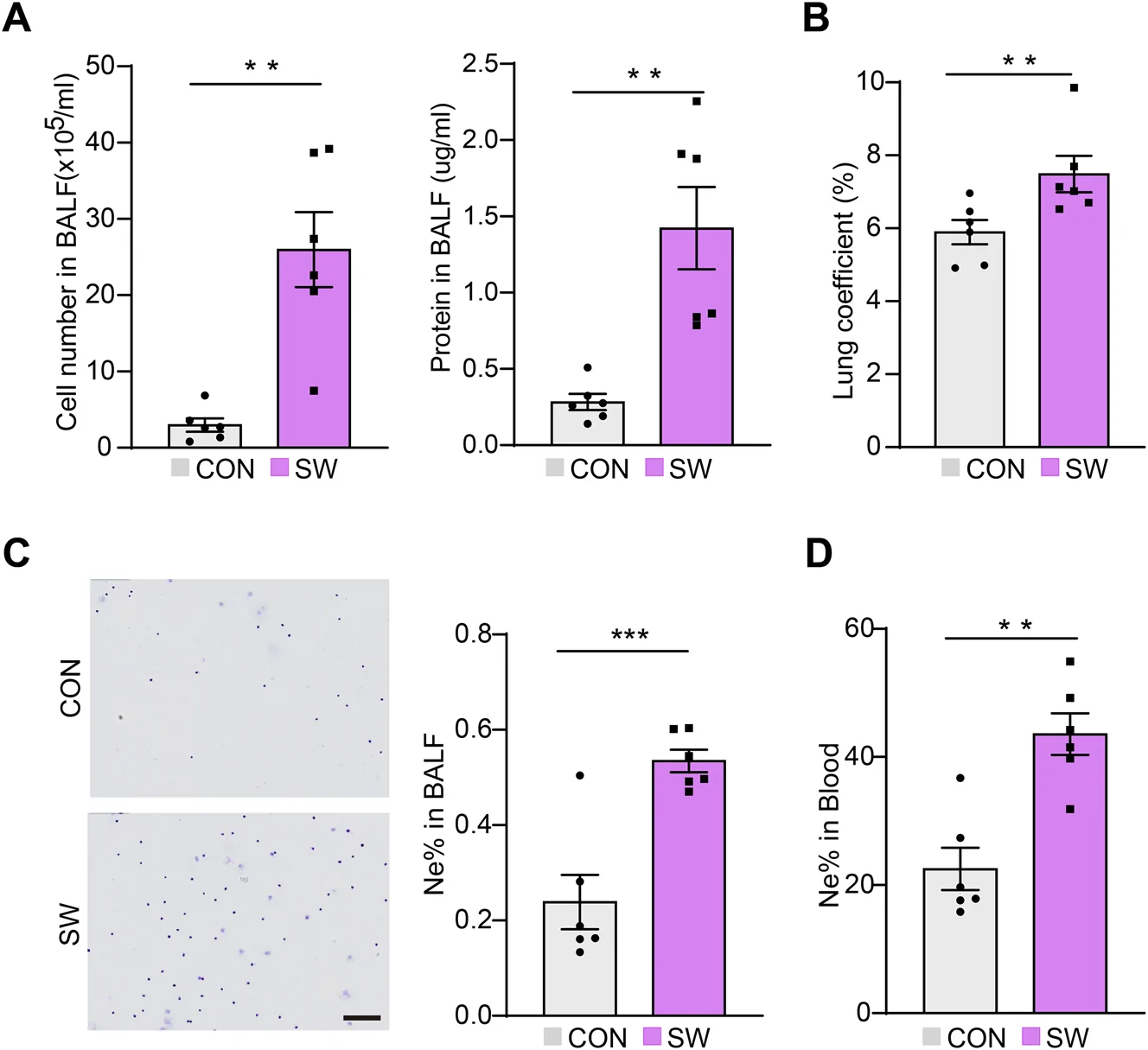
The LEI method of preparing a mouse SWD-ALI model conforms to the latest standards. Notes: A Total cell counts and total protein concentrations in the BALF; B mouse lung coefficient; C H&E staining images of BALF cells, and quantitative analysis of the NE% in BALF; D quantitative analysis of the NE% in blood. n=6, **p<0.01, ***p<0.001.

From the above results, it was confirmed that the model mice showed obvious lung histologic damage (n=6 per group). We then examined cell and protein exudates in BALF (Figure 6A) and calculated lung coefficients (Figure 6B) indicating altered permeability of the lung air-blood barrier. It was found that the mice in the model group showed significant air-blood barrier permeability damage (p<0.05). The neutrophil count in BALF was markedly elevated in the model group (p<0.05) (Figure 6C), which represented an enhanced inflammatory response. A significant increase in peripheral blood neutrophils was also observed in the model group (Figure 6D), combined with clinical symptoms such as cyanosis and dyspnea, which were consistent with physiological dysfunction [19]. Therefore, the mouse SWD-ALI model, prepared by LEI of 4 mL/kg artificial seawater after 6 h, fully meets the “four core criteria for animal ALI” proposed by the American Thoracic Society official workshop report [19].

## Discussion

In the present study, we found that the laryngoscopic endotracheal injection (LEI) method was able to establish a SWD-ALI mouse model with moderate mortality, controllable injury severity, and good clinical simulation; micro-CT can accurately identify the lesion area and evaluate the severity of SWD-ALI in the early stage (as early as 0.5 h post-injury), and the quantitative indices of micro-CT are significantly correlated with pathological injury scores, which can provide a reliable basis for early clinical treatment guidance. The sample size and experimental design of this study are consistent with our previously published SWD-ALI research [17], with core results (inflammatory factor upregulation, lung coefficient elevation, micro-CT imaging changes) successfully reproduced, further supporting the validity and reproducibility of the experimental findings. SWD-ALI is a complex pathological process caused by direct hypertonic injury of seawater to the lung tissue and subsequent severe inflammatory response [7], with an extremely high mortality rate due to the lack of effective early assessment and treatment methods [6]. We systematically explored the effects of various modeling approaches, artificial seawater doses and injury time on SWD-ALI induction in mice, and clarified the value of micro-CT in early injury assessment, which provides an important experimental basis for in-depth study of SWD-ALI pathogenesis and clinical translation.

Based on previous studies, we selected three commonly used methods to prepare ALI models and innovatively applied the LEI method for comparison [7], [8], [9, 20]. Compared with the control mice, the mice of all four modeling methods showed different degrees of choking, shortness of breath, white foam overflow, and abnormalities in respiratory rate, activity, and diet. Among them, the ND method was easy to operate, but the performance of mice was the lightest, and the dose of seawater inhalation was not controllable, leading to poor model stability. The DD method could simulate the real drowning situation well, but the mortality rate was extremely high (85.7 % within 24 h), which not only leads to small effective sample size but also increases animal suffering, which is violated the 3R principles the 3R principle. The NEI method has a relatively stable injury effect but requires invasive neck incision, which causes severe systemic inflammatory response and long anesthesia time, leading to secondary injury and inconsistent with the clinical characteristics of seawater drowning. In this study, we proposed the LEI method for the first time to prepare SWD-ALI models, which highly simulated the real drowning scenarios (seawater inhalation via the upper respiratory tract), avoided the neck incision, and the operation was faster and easier by comparison (Table 1). In addition, this method uses isoflurane inhalation anesthesia with short duration and rapid awakening, avoiding the effect of prolonged anesthesia on mice, and the moderate mortality rate (28.6 % within 24 h) ensures sufficient effective sample size for subsequent mechanistic and therapeutic studies – ” a key advantage for studies of pathologic mechanisms and for use with genetically altered animals”.

**Table 1:** Comparison of different SWD-ALI mice modeling methods.

Methods	Operation steps	Advantages	Disadvantages
Direct drowning (DD)	1. Place the mouse in a plastic container filled with seawater, allowing the mouse to be immersed in seawater; 2. Immerse for 25 s, then pat dry and observe	Simulates a real sense of suffocation, relatively quick and convenient	Significant individual differences in mouse, extremely high mortality rate
Nasal drip (ND)	1. Inject a certain amount of seawater into the mouse’s nasal cavity; 2. Hang the mouse for 15 s and observe the drowning process	Convenient operation	Relatively poor reliability of inhaled dose, some may be swallowed
Neck incision endotracheal injections (NEI)	1. Perform anterior tracheotomy; 2. Inject seawater into the incision; 3. Hang for 15 s and observe the drowning process	Highly realistic, relatively stable	Slightly difficult operation, slightly longer anesthesia time, higher systemic inflammatory response
Oral laryngoscope endotracheal injection (LEI)	1. Lift the epiglottis under a laryngoscope and observe the opening and closing of the glottis with breathing; 2. Inject a certain amount of seawater with a microliter syringe; 3. Hang for 15 s, and observe the drowning process	Highly realistic, requires short anesthesia time, relatively stable, moderate mortality	Slightly difficult operation, repeated operations may cause passive injury to mouse

To verify the compliance with the criteria after modeling, we based our criteria on the latest animal ALI model proposed by the official workshop report of the American Thoracic Society [19], which is featured in four aspects: tissue damage, alteration of alveolar-capillary barrier, inflammatory response, and physiological dysfunction. In this study, the lungs of the model group revealed obvious tissue damage including alveolar cell injury, alveolar septal thickening/edema, intra-alveolar hemorrhage, and alveolar structure destruction, with clear evidence of histological damage [21], [22], [23]. The marked elevation in lung coefficient and BALF total protein concentration suggested impaired alveolar-capillary barrier integrity [24], [25], [26]. The percentage of neutrophils in BALF and peripheral blood of mice was significantly elevated, revealing a severe inflammatory response after seawater drowning [7], 27], 28]. Clinical symptoms such as cyanosis, bradypnea and reduced activity in SWD-ALI mice suggested obvious physiological dysfunction [5], 18], 29], 30]. In summary, SWD-ALI mouse model prepared by LEI fully conforms to the international standard of animal ALI models, with good validity and clinical relevance.

Micro-CT is a specialized type of CT that is commonly used for imaging examinations of small animals. This study also used CT, a common clinical imaging examination [31], 32], to evaluate early lung injury in SWD-ALI mice, and found that it could quickly identify and assess the severity of injury in the early stage. Marked exudative changes were observed as early as 0.5 h after injury, and exudation peaked at 2 h, with significantly increased average gray value and percentage of damaged area in lung CT, and even “white lung” like changes in severe cases – consistent with the early imaging characteristics of clinical ALI/ARDS [18]. We further validated the accuracy of micro-CT by Pearson correlation analysis, and found a significant positive correlation between micro-CT damaged area percentage and pathological total injury score (r=0.64, p<0.001), which verified that micro-CT can accurately quantify the severity of SWD-ALI.

However, the results of pathological and molecular tests of lung tissue injury were not consistent with imaging results, with the pathological peak lagging behind the imaging peak (imaging peak at 2 h, pathological peak at 6 h). This experiment suggested that the early stage of SWD-ALI in mice is dominated by the exudation of small molecules such as water and electrolytes, due to hypertonic stress, which leads to a sharp increase in imaging damage (CT is highly sensitive to liquid exudation [32]). With the aggravation of the disease, the exudate is gradually transformed into proteins and inflammatory cells, while the water is reabsorbed by the body, so the imaging results suggest an improvement, but the pathological results show that inflammatory cells and other substances are continuously exuding into the alveolar space, leading to the continuous aggravation of pathological injury. Most SWD-ALI mice were significantly aggravated or died in the early stage, so early treatment is the key to affect the prognosis [6]. Micro-CT can quickly identify SWD-ALI in the early stage, and with the clinical application of mobile CT detection means, timely CT detection can directly guide early treatment options (such as reducing exudates, removing airway secretions, or emphasizing lung ventilation) and affect the early mortality and prognosis of drowning patients.

The artificial seawater used in this study was formulated based on the ionic composition of seawater along the southeast coast of China, with a total salinity of 35 g/L (consistent with the average global ocean salinity of 33–37 g/L) [3]. Regional differences in seawater composition are mainly reflected in the slight variation of major cation/anion concentrations (e.g., higher Mg^2+^/Ca^2+^ in the Pacific Ocean vs. the Atlantic Ocean [3]) and the content of trace elements (e.g., iodine, strontium) and organic matter, while the core hypertonic components (NaCl, Mg^2+^, Ca^2+^ salts) that induce SWD-ALI remain consistent across global oceans. The hypertonic stress caused by 3.5 % NaCl is the primary driver of alveolar capillary fluid shift and pulmonary edema in SWD-ALI [7], and the Mg^2+^/Ca^2+^ in the artificial seawater mimics the pro-inflammatory and endothelial damage effects of natural seawater [16] – these core pathogenic factors are retained in our formulation. Although seawater collected from different regions may exhibit slight variations in trace element composition, previous studies have confirmed that trace components have no significant effect on the overall pathological process of SWD-ALI, and the hypertonic-induced ALI is the dominant pathological change [7]. Therefore, our LEI-based SWD-ALI model, established with southeast China coastal seawater components, can reflect the core pathological characteristics of SWD-ALI in global marine regions. For studies focusing on regional seawater-specific injury mechanisms (e.g., high trace metal content in coastal industrial areas), the artificial seawater formulation can be slightly adjusted based on local seawater ion detection data, and the LEI modeling method remains applicable with good reproducibility.

The LEI model established in this study has good translational relevance to clinical ALI, as it mimics the clinical characteristics of seawater inhalation via the upper respiratory tract, avoids invasive surgery, and the injury severity and recovery course are consistent with clinical patients [5]. This model can be used to study the core pathogenic mechanisms of SWD-ALI (e.g., hypertonic stress-induced ferroptosis [16], inflammatory response [7]) and screen potential therapeutic drugs. Bora SE et al. [33]. found that octreotide has a short-term protective effect on sepsis-induced lung injury in rats by inhibiting inflammatory response and reducing lung vascular permeability, which provides an important reference for the treatment of SWD-ALI. Since SWD-ALI and sepsis-induced lung injury share similar pathological processes (severe inflammatory response and alveolar-capillary barrier damage [28]), octreotide may also have a protective effect on SWD-ALI, which is worthy of further study using the LEI model. In addition, our study found that micro-CT can accurately evaluate early lung injury in SWD-ALI mice, which is consistent with the clinical application of CT in the diagnosis of drowning-induced lung injury [32], and provides an experimental basis for the clinical use of mobile CT for early bedside assessment of SWD-ALI patients.

Long-term observations in this study showed that model mice exhibited bronchiolar epithelial shedding (3 days), blunt rupture (7 days), and subpleural alveolar fusion (28 days) post-injury, which are potential pathological changes leading to chronic lung injury [22]. It is not clear whether these changes are related to bronchiolar remodeling [34], 35], and further studies are needed to determine whether SWD-ALI mice develop chronic changes such as emphysema [36], 37]. Therefore, we need to adopt effective evaluation tools (such as micro-CT) during the golden period of rescue to guide targeted therapy, which would not only reduce the severity of lung injury in the acute phase, but also reduce the occurrence of chronic lung changes and improve the long-term prognosis of SWD-ALI patients.

## Conclusions

The “laryngoscopic endotracheal injection (LEI)” method of artificial seawater can successfully establish the SWD-ALI model in mice with a high degree of simulation, stable injury severity, and moderate mortality, and the operation is quicker and simpler – this model is suitable for in-depth study of SWD-ALI pathogenesis and therapeutic drug screening. Lung micro-CT can quickly detect and quantify SWD-ALI damage in the early stage (as early as 0.5 h post-injury), accurately evaluate the severity of lung injury, and determine the golden period of treatment for severe SWD-ALI patients, which can guide clinical treatment and attenuate the acute severity and long-term prognosis of patients.
